# A Comparative Analysis of the Impact of Two Different Cognitive Aid Bundle Designs on Adherence to Best Clinical Practice in Simulated Perioperative Emergencies

**DOI:** 10.3390/jcm13175253

**Published:** 2024-09-05

**Authors:** Maartje van Haperen, Tom C. P. M. Kemper, Lena Koers, Suzanne B. E. van Wandelen, Elbert Waller, Eline S. de Klerk, Susanne Eberl, Markus W. Hollmann, Benedikt Preckel

**Affiliations:** 1 Department of Anaesthesiology, Amsterdam University Medical Centre, 1105 AZ Amsterdam, The Netherlands; m.vanhaperen@amsterdamumc.nl (M.v.H.); t.kemper@amsterdamumc.nl (T.C.P.M.K.); sbevanwandelen@gmail.com (S.B.E.v.W.); e.waller@amsterdamumc.nl (E.W.); e.s.deklerk@amsterdamumc.nl (E.S.d.K.); m.w.hollmann@amsterdamumc.nl (M.W.H.); 2 Department of Paediatric Intensive Care, University Medical Centre Leiden, 2333 ZA Leiden, The Netherlands; lena.koers@gmail.com

**Keywords:** cognitive aid bundle, emergency manual, cognitive aid design, simulated perioperative emergency

## Abstract

**Background:** Stress and human error during perioperative emergency situations can significantly impact patient morbidity and mortality. Previous research has shown that cognitive aid bundles (CABs) minimize critical misses by 75%. This study aimed to compare the effectiveness of two different CAB designs with the same content in reducing missed critical management steps for simulated perioperative emergencies. **Methods:** A multicenter randomized controlled simulation-based study was conducted including 27 teams, each consisting of three participants; each team performed four simulation scenarios. In the first scenario for each team (Scenario 1), no CAB was used. Scenarios 2 and 3 were randomly allocated to the groups, with either a branched, clustered design (CAB-1) or a linear, step-by-step design (CAB-2) of the cognitive aid. In Scenario 4, the groups used one of the previously mentioned CABs according to their own preference. The primary outcome was the difference in the percentage of missed critical management steps between the two different CABs. Secondary outcomes included user preference for one CAB design and the reduction in percentage of missed critical management steps using any CAB versus no CAB. **Results:** Twenty-seven teams simulated 108 perioperative emergency situations. The percentage of missed critical management steps was similar between CAB-1 and CAB-2 (27% [interquartile range (IQR) 20–29] versus 29% [IQR 20–35], *p* = 0.23). However, most participants favored the branched, clustered design CAB-1 (77.8%). Additionally, employing any CAB reduced the percentage of missed critical management steps by 36% (33% missed steps vs. 21% missed steps, *p* = 0.003). **Conclusions:** While the two CAB designs did not differ significantly in reducing missed critical management steps, the branched, clustered design was perceived as more user-friendly. Importantly, using any CAB significantly reduced the percentage of missed critical management steps compared to not using a cognitive aid, emphasizing the need for CAB use in the operating room.

## 1. Introduction

Human fallibility in perioperative emergencies can result in preventable patient harm and thus increased healthcare costs [1,2]. To reduce human error in critical situations, cognitive aid bundles (CABs) have been developed, guiding users when performing complex tasks and improving team performance. Employing CABs reduces the number of missed critical management steps during simulated perioperative emergencies by up to 75% [3,4,5].

The design of CABs influences usability and may even contribute to human error [6,7,8,9]. While most studies have focused on the impact of the content of CABs, fewer studies have looked at the impact of the design of the bundles. Marshall et al. showed superior team performance when using a linear versus a branched design for CABs [7]. Another study reported no effect on clinical performance when comparing paper versus digital CABs [10], although users preferred the paper version [11]. Which design features of CABs have the most impact on adherence to best clinical practice is still a matter of debate.

In this study, we compared the effectiveness of two different CABs in reducing the percentage of missed critical management steps in simulated crisis scenarios. Both CABs had the same content but a completely different design. We expected that a branched, clustered CAB design would lead to a greater reduction in the percentage of missed critical management steps than a linear, step-by-step CAB design.

## 2. Materials and Methods

In this multicenter prospective randomized controlled study, two different CABs with the same content but a completely different design were compared with the aim of reducing the percentage of missed critical management steps in simulated crisis scenarios.

After reviewing the trial protocol, the Medical Ethics Review Committee of the Amsterdam University Medical Center (UMC) (Ethical Committee No. NAC 207) provided a waiver under the Medical Research Involving Human Subjects Act (W19_189#19.228). Written informed consent was obtained from all participants. This study was registered in the Dutch Trial Register (NTR-NL8354) and conducted in compliance with the Declaration of Helsinki; the writing of this report adhered to the CONSORT guidelines [12].

### 2.1. Participants

Participants were divided into fixed teams of three members, based on their clinical practice experience. Each team required a minimum of one anesthesiologist and one nurse anesthetist, along with either an extra nurse anesthetist, a scrub nurse, or anesthesia resident. The participating team members were included based on availability, willingness, and Dutch language skills and had to work in an operating room (OR). Only participants not familiar with the two CABs were enrolled.

### 2.2. Design of the Cognitive Aid Bundles

**Figure 1:**
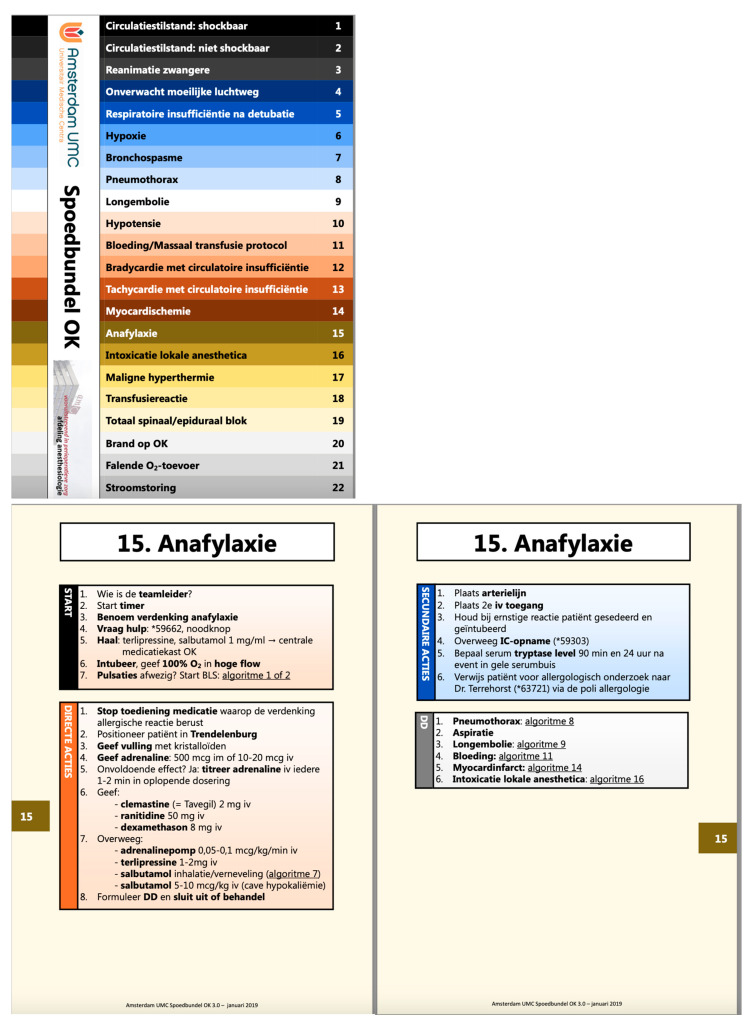
A branched, clustered cognitive aid bundle (CAB) design (CAB-1, University Hospital of Amsterdam) with a color-coded table of contents on the front cover (**top**) and corresponding side tabs to guide the user to the 22 algorithms (**bottom**). The individual algorithms (e.g., anaphylaxis) are all constructed with clear grouped text blocks, including ‘Start items’ at the top of the left page, followed by Direct actions’ and ‘Secondary actions’. A ‘Differential diagnosis’ or other information may appear on the right page in some algorithms.

**Figure 2:**
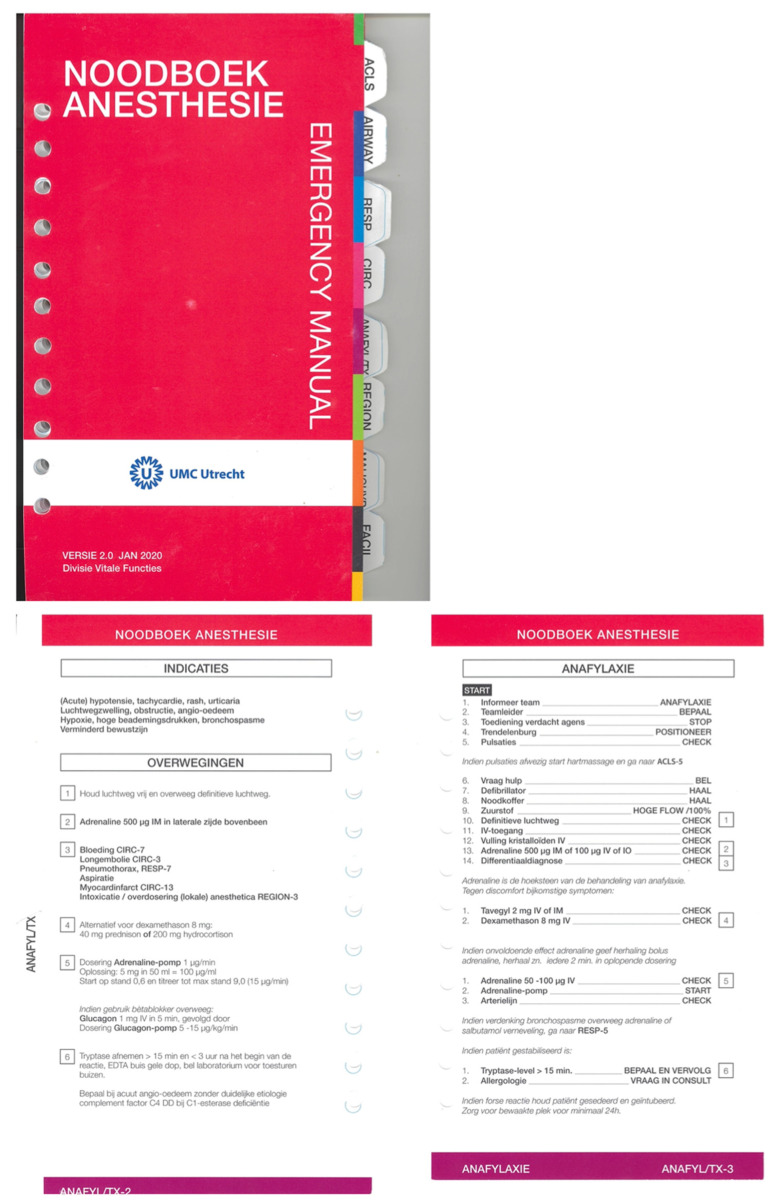
A linear, step-by-step cognitive aid bundle (CAB) design (CAB-2, University Hospital of Utrecht), with a bright red front cover (**top**), was developed in collaboration with the Dutch Royal Air Force and is based on their extensively evaluated Chinook CH-47D/F helicopter emergency checklist. White side tabs direct readers to each of the ABCDE-structured chapters (**bottom** of figure). A table of contents introduces each algorithm (e.g., anaphylaxis). At the bottom of each page, vertical tabs help readers find the correct algorithm. On the right page of the CAB, each algorithm begins with steps, evaluations, and actions. For more detailed information on different steps, the reader is directed to the corresponding paragraph on the left-hand page, indicated by numbers.

Both CAB-1 and CAB-2 used exactly the same content, which was derived from the Stanford bundle of cognitive aids for operating room emergencies [13,14] and adapted to the local setting in two university hospitals in The Netherlands (CAB-1, University Hospital of Amsterdam; CAB-2, University Hospital of Utrecht). However, the CABs had completely different designs. The key difference in the designs is that CAB-1 has a branched, clustered CAB design, with a clearly visible table of contents on the front page and text blocks that are grouped together (clustered, Figure 1). CAB-2 has a linear, step-by-step CAB design that follows the ABCDE structure and employs a more sequential arrangement of text (Figure 2).

### 2.3. Setting

This study was conducted between May 2019 and February 2023 at three Dutch hospitals that were carefully chosen based on their accessibility and lack of familiarity with either one of the two CABs used in the study (Franciscus Gasthuis & Vlietland, Van Weel-Bethesda, Spijkenisse Medisch Centrum). A designated study coordinator at each hospital was tasked with obtaining permission to use their facilities as needed for in situ simulations. Therefore, the coordinators received detailed information on the purpose of the study upfront, were briefed on the necessary logistics, and were asked to find local volunteers. The participants signed their formal informed consent on the day the simulation scenarios were performed, but before participation.

The study was conducted within the respective hospital ORs, using in situ simulation to replicate perioperative emergencies. A standardized setup was used with the Laerdal SimMan^®^ 3G manikin. For data acquisition, all simulation sessions were audiovisually recorded within the Laerdal SimView (Laerdal, Wappingers Falls, NY, USA). All recordings were collected, stored, and processed according to Good Clinical Practice [15].

The simulation was run by a certified operator, who selected the scenario from five pre-programmed OR emergencies: bronchospasm, anaphylactic shock, massive hemorrhage, ventricular fibrillation (VF), and pulseless electric activity (PEA). Shortly before the simulation session, team members were provided with detailed information on the study’s purpose. Thereafter, participants were given five minutes to familiarize themselves with both CABs and the setup with the Laerdal SimMan^®^ 3G manikin. The team received a written briefing about the preoperative assessment of the simulated patient and the scenario settings, including surgery progress and timeline. The team was advised to have a reader reading out the respective algorithm, with a different team member starting the subsequent scenarios. The teams were given ten minutes for each scenario. After the fourth scenario, the team received a short debrief of the simulated sessions.

### 2.4. Outcome Parameters

The primary parameter of this study was the percentage of missed critical management steps in simulated crisis scenarios with a branched, clustered CAB design (CAB-1) compared to a linear, step-by-step CAB design (CAB-2). The secondary parameters were the perceived user-friendliness of the CABs, based on team preference for one of the two CABs during the last scenario, and the percentage of missed critical management steps when using any CAB versus no CAB.

### 2.5. Randomization

Each team participated in four simulated perioperative emergency scenarios, in which the percentage of missed critical management steps was determined. Scenario 1 was conducted without the use of a CAB, employing one of two different perioperative advanced cardiac life support (ACLS) emergencies, specifically either VF or PEA. This inherently implied that the other option (not used in Scenario 1) was used in Scenario 4 for the respective group. The purpose of this initial scenario was to familiarize the teams with the setup and minimize the impact of the learning process.

For the second and third scenarios, the teams were randomly assigned to CAB-1 or CAB-2 and to two of the three remaining perioperative emergency options (e.g., bronchospasm, anaphylaxis, or massive bleeding). Randomization was achieved by a team member blindly drawing tickets from a sealed, opaque envelope containing the assignments for both the CABs and the scenarios.

In the final scenario (Scenario 4), the teams were allowed to select their preferred CAB. The type of perioperative ACLS emergency was predetermined by the choice/allocation in Scenario 1.

As in a previous study, we used 15 pre-defined essential management steps for all scenarios, based on best practice recommendations from literature; see Supplement S1 [4]. An independent observer scored the adherence rate to these essential management steps per CAB in a binary or ternary way, employing the pre-defined scoring sheets. Scoring was conducted offline using the video recordings of the sessions. A critical step completed outside the specified time frame received 1 point instead of 2 points. Due to the nature of the study, blinding the assessor during the scoring of these sessions was not possible. Total scores were converted into percentages of the maximum achievable score in each single scenario to compare different scenarios with each other.

### 2.6. Sample Size

To determine the optimal number of teams required to assess the effectiveness of different CAB designs, we conducted a pilot study. Our pilot study revealed an error rate of 1.5 (2% versus 14%) between the two CABs. To account for a potential learning effect and to achieve a power of 90%, a sample size of 25 teams was required. This was determined based on a baseline fault rate of 10% when using CAB-1 and the need to detect a 5% difference (10% vs. 15% missed management steps) between the groups, with a factor of 1.5 [4]. A two-sided Z-test for two means was used, with an alpha level of 0.025. Consequently, this resulted in a sample size of 25 teams. However, to account for any potential technological complications and dropouts, our objective was to include 27 teams.

### 2.7. Statistics

Statistical analysis was performed using IBM SPSS Statistics version 26.0 (IBM Corp., Armonk, NY, USA) for Windows. A *p*-value < 0.05 was considered statistically significant. The Kolmogorov–Smirnov test was used to check the distribution of data. Data are presented as mean ± standard deviation (SD) or median with interquartile range (IQR), depending on the distribution of the data. To compare the between-group differences in failure of adherence to best clinical practice, the Mann–Whitney U test was used. Descriptive statistics were used to evaluate the characteristics and the perceived usability of the CABs.

## 3. Results

**Figure 3:**
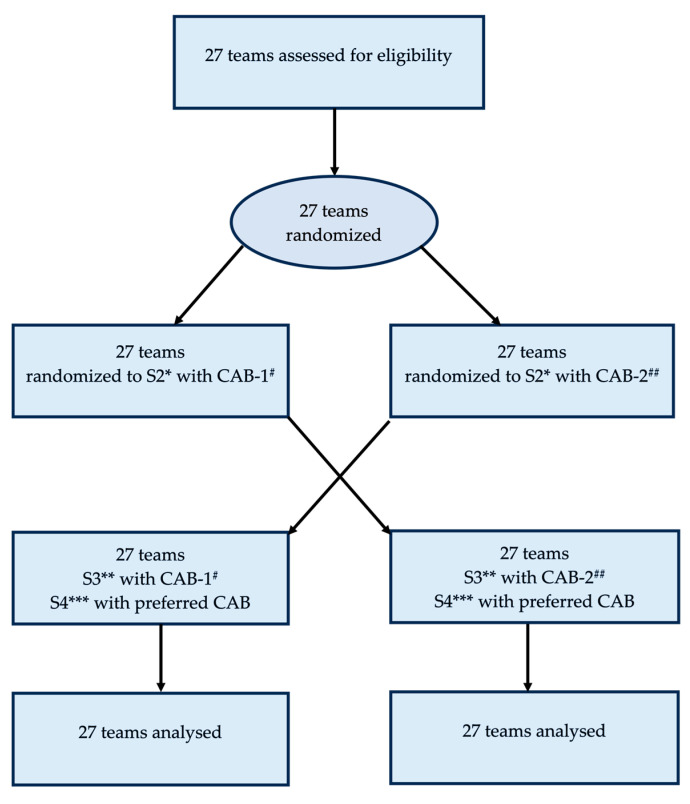
CONSORT flow diagram. Abbreviations: * S2 = scenario 2, ** S3 = scenario 3, *** S4 = scenario 4 and ^#^ CAB-1 = cognitive aid bundle 1, ^##^ CAB-2 = cognitive aid bundle 2.

Twenty-seven anesthesia teams consisting of 81 participants (27 consultant anesthesiologists, 38 anesthesia nurses, 5 scrub nurses, 2 post-anesthesia care unit nurses, and 9 junior anesthesia nurses) participated in four simulated perioperative emergency scenarios per team. The consort flow diagram is shown in Figure 3. There were no dropouts.

The CABs and the simulated emergency situations were evenly distributed across the scenarios.

In Scenario 2, the teams started 15 times with CAB-1 (a branched, clustered design) and 12 times with CAB-2 (a linear, step-by-step design). The simulated emergency situations included nine cases of anaphylaxis, seven cases of bronchospasm, and eleven cases of massive hemorrhage.

In Scenario 3, the teams started with the other CAB (not used in Scenario 2); thus, they started 12 times with CAB-1 and 15 times with CAB-2. The simulated emergencies during Scenario 3 comprised nine cases of anaphylaxis, nine cases of bronchospasm, and nine cases of massive hemorrhage.

For Scenario 4, the teams could choose which CAB they would like to use based on the experience in the previous scenarios: CAB-1 was used twenty-one times and CAB-2 six times. During Scenario 4, the teams had to deal 15 times with VF and 12 times with PEA.

A median of 27% [IQR 20–29] of the critical management steps were missed while using CAB-1, compared to 29% missed steps [IQR 20–35] while using CAB-2. There was no significant difference in the percentage of missed critical management steps between CAB-1 and CAB-2 (*p* = 0.23).

CAB-1 was perceived to be more user-friendly; 21 (78%) of the participating teams preferred its use for the last scenario.

The percentage of missed critical management steps using any CAB versus no CAB resulted in a significant decrease in missed critical management steps: 36% (a median of 33% [IQR 26–53] missed steps versus 21% [IQR 16–35] missed steps), *p* = 0.003.

## 4. Discussion

**Figure 4:**
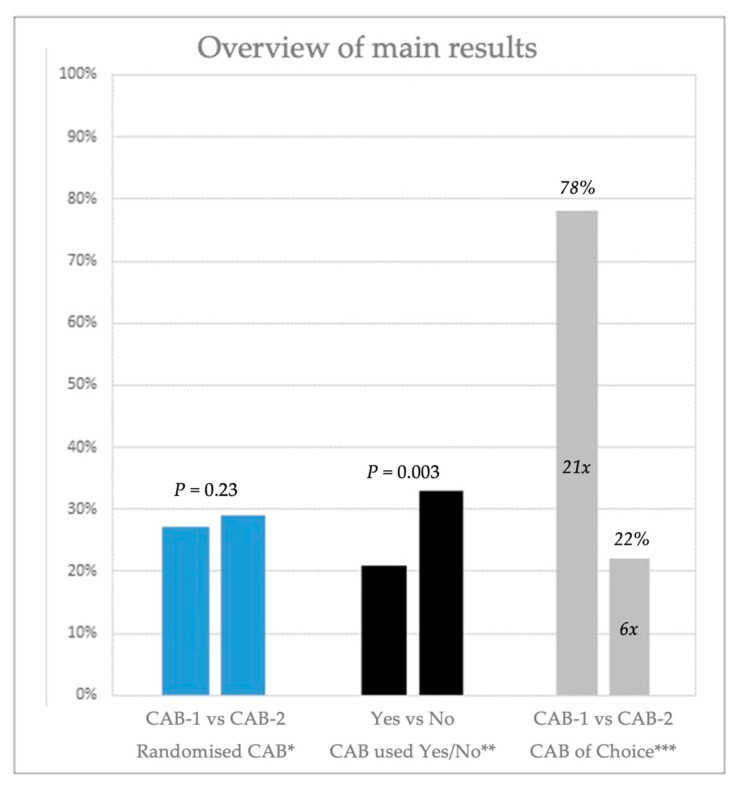
Summary of main results; * percentage of missed critical management steps, ** percentage of missed critical management steps, and *** percentage of which cognitive aid bundle (CAB) was perceived as more user-friendly.

In this study, we compared two CABs with the same content but different designs. More specifically, we compared a branched, clustered CAB design (CAB-1) with a linear, step-by-step CAB design (CAB-2) and looked at the percentage of missed critical management steps in simulated crisis scenarios. In contrast to our hypothesis, we did not find a difference in the percentage of missed critical management steps between the two differently designed CABs. However, we found a significant reduction in the percentage of missed critical management steps using any of the CAB versus not using a CAB (Figure 4). A branched, clustered CAB design was chosen more frequently and was perceived as more user-friendly.

Our study is the first to directly compare a branched, clustered CAB design and a linear, step-by-step CAB design containing exactly the same content on adherence to best practice in a simulated emergency setting. Previous studies demonstrated improved team performance with a linear CAB design based on the execution of non-technical skills, the timing of onset, and the execution of important critical management steps during simulated allergic reactions [7]. In contrast, by measuring eye tracking of participants, it was shown that using a branched, clustered CAB design resulted in faster information retrieval than a traditional step-by-step design [16]. However, the connection between looking for specific information (measured by eye tracking) and the subsequent performance of a critical management step was not included in their analysis [16]. Although previous research suggests that certain CAB designs may be more effective for specific aspects, e.g., team function and faster information retrieval, our study demonstrated that the design of a CAB does not have a significant influence on adherence to best clinical practice (Figure 4). It is important to realize that the precise impact of an individual missed critical management step on patient outcome is still unknown. Nevertheless, as the critical management steps are derived from best clinical practice, it is likely that fewer management steps missed in an emergency could eventually result in an improved patient outcome.

It might be that the simulated scenarios employed in the present study focused on critical management steps that were very familiar to the participating healthcare professionals; thus, they might not have missed a significant number of critical steps at all, independently from using the CAB. However, when comparing those scenarios where any CAB was used to those not using a CAB, we clearly confirmed previous findings [3,4] that using a CAB significantly reduces missed management steps in simulated emergency scenarios. Using rarer emergency situations (e.g., malignant hyperthermia, tension pneumothorax, etc.) might therefore reveal differences in CAB design missed in our present study.

Training healthcare professionals to familiarize them in the use of a CAB during emergency situations is essential for determining the appropriate time and method to properly use the respective CAB [5]. While a CAB should ideally be user-intuitive, training with each of the CABs used in our study might have been preferable. However, as we made sure that the participants were not familiar with any of the CABs used, we can at least exclude any experience effect in using the specific bundles from the present investigation.

Our study participants were multidisciplinary operating room teams with different clinical experience levels who were unfamiliar with the simulation modality. It is known that using simulation training is able to improve the quality and safety of care [17], and performing the same study with participants already exposed to simulation training might reveal different results, so the benefits of using a specific CAB may have been underestimated in our study. Our main outcome parameter was calculated based on data from Scenario 2 and Scenario 3. Thus, the teams were able to familiarize themselves with collaborating in a simulation environment in Scenario 1, thereby reducing the influence of a learning process during simulation training on the primary outcome results.

Our secondary outcome clearly showed that most participants perceived a branched, clustered CAB design to be more user-friendly, which corresponds with the results from previous studies [8,16]. An effective CAB should support both “sampling” and a step-by-step guided workflow. Although a CAB should ideally be intuitive and suitable for individuals with varying levels of expertise, novices tend to gain more information from a systematic approach (a step-by-step guide), while specialists are more inclined to seek out specific information (sampling) [16,18,19]. Realizing that higher usability of a CAB most likely results in a higher usage of the CAB in perioperative emergencies, it is important to define in future studies which design features increase the usability of a CAB [4,20,21].

## 5. Limitations of the Study

The goal of our study was to compare two CABs with the exact same content but different structures. It was not our intention to compare different bundles with different contents. Both bundles used in our study were developed at the same time and were based on the Stanford Emergency Manual. The content was exactly the same, giving us a unique chance to compare quite different bundles (structure) with the same content. We therefore cannot draw conclusions about other available bundles and guidelines.

As mentioned before, using emergency scenarios that occur more rarely during clinical practice might have revealed differences in missed steps, as in those cases, the knowledge for the rare scenario might be lower than for scenarios used in the current study.

## 6. Conclusions

This study found no difference in adherence to best clinical practice between a branched, clustered CAB design and a linear, step-by-step CAB design with the exact same content. Using any CAB design improved adherence to best clinical practice compared to not using a CAB. Most participants reported that a branched, clustered CAB design was easier to use; expecting that clinicians are more likely to use a CAB during perioperative emergencies if it is user-friendly, it might be reasonable to choose a branched, clustered CAB design in the future.

## Data Availability

Data will be made available to researchers on due request.

## Supplementary Materials

The following supporting information can be downloaded at https://www.mdpi.com/article/10.3390/jcm13175253/s1, Supplement S1: Scoring sheets of the critical management steps for the scenarios: anaphylaxis, bronchospasm, massive bleeding, pulseless electric activity (PEA) without bundle, pulseless electric activity (PEA) with bundle, ventricular fibrillation (VF) without bundle and ventricular fibrillation (VF) with bundle.
