# Supplementary material for: A Comparative Analysis of the Impact of Two Different Cognitive Aid Bundle Designs on Adherence to Best Clinical Practice in Simulated Perioperative Emergencies
Source: J Clin Med. 2024 Sep 5;13(17):5253. doi: 10.3390/jcm13175253 (PMC11395788; doi:10.3390/jcm13175253)
Supplement: Supplementary file 1 [file jcm-13-05253-s001.zip › jcm-3155525-supplementary.pdf]

Supplement S1 Scoring sheets of the critical management steps

Scenario Anaphylaxis

Session No.:

Investigator:

Date:

Bundle:

Act	Performed<br>0 = no 1 = yes<br>2 = within time note	Remark
1. Asks for help		
2. Informs team		
3. Names problem		
4. Oxygen 100% high flow		
5. Discontinues medication on which suspected allergic reaction within 2 minutes		
6. Check pulsations		
7. Positions patient in Trendelenburg		
8. Administer adrenaline < 5 minutes		
9. Checks airway		
10. Administers fluid bolus		
11. Nebulizes with salbutamol		
12. Clemastine 2 mg IV		
13. Hydrocortisone (steroid) 100 mg IV		
14. Sampling lab including tryptase		
15. Clear division of roles, including team leader		

Total score	
Duration scenario	
Duration until maximum score	

Supplement S1 Scoring sheets of the critical management steps

Scenario Bronchospasm

Session No.:

Date:

Investigator:

Bundle:

Act	Performed<br>0 = no 1 = yes<br>2 = within time note	Remark
1. Asks for help		
2. Informs team		
3. Oxygen 100% high flow		
4. Controls position and depth tube		
5. Adrenaline 10 mcg IV		
6. Sucking out tube		
7. Administer salbutamol puffs or nebulization		
8. Increase expiration time		
9. DD/ consider anaphylaxis, auto PEEP		
10. Consider vaporous anesthetics		
11. Consider adrenaline nebulization 5 ml (100mcg/ml)		
12. Salbutamol 5-10 mcg IV		
13. Magnesium 2 gr IV		
14. Take bloodgas		
15. Clear division of roles , including team leader		

Total score	
Duration scenario	
Duration until maximum score	

Supplement S1 Scoring sheets of the critical management steps

Scenario Massive Bleeding

Session No.:

Investigator:

Date:

Bundle:

Act	Performed<br>0=no 1=yes<br>2=within time note	Remark
1. Asks for help		
2. Informs team		
3. Oxygen 100% high flow		
4. Activates MTP		
5. Stop the bleeding (surgeon)		
6. Positions patient in Trendelenburg		
7. Gives large IV access		
8. Gives tranexamic acid 1 gr IV		
9. Accept MAP 55, give fluids and vasopression only if necessary		
10. Give bloodproducts		
11. Check for blood thinners to antagonize		
12. Check Lab		
13. Target $Ca^{2+} > 1$ mmol/L		
14. Target normothermia T > 36° C		
15. Clear division of roles, including team leader		

Total score	
Duration scenario	
Duration until maximum score	

Supplement S1 Scoring sheets of the critical management steps

Scenario PEA without bundle

Session No.: **Investigator:**

Date: **Bundle:**

Act	Performed<br>0=no 1=yes<br>2=within time note	Remark
1. Checks pulsations within 1 min		
2. Starts BLS within 1 min of detecting circulatory arrest		
3. Checks electrodes/ monitor settings		
4. Asks for help		
5. Starts timer		
6. Good quality BLS (5-6 cm deep, 100-120 x p/m) / 30:2		
7. Increases FIO2 to 100%		
8. Checks IV-access		
9. Paused BLS < 15 seconds		
10. Correctly connects defibrillator < 2 min from circulatory arrest		
11. Identifies non-shockable rhythm		
12. Adrenaline (1 mg) is given IV in first block		
13. Rhythm check/ for pulsations every 2 minutes		
14. DD/ is named aloud using 4H/ 4T.		
15. Clear division of roles, including team leader		

Total score	
Duration scenario	
Duration until maximum score	

Supplement S1 Scoring sheets of the critical management steps

Scenario PEA with bundle

Session No.:

Date:

Investigator:

Bundle:

Act	Performed<br>0=no 1=yes<br>2=within time note	Remark
1. Controls pulsations within 1 min		
2. Starts BLS within 1 min of detecting circulatory arrest		
3. Uses bundle within 2 minutes		
4. Asks for help		
5. Starts timer		
6. Good quality BLS (5-6 cm deep, 100-120 x p/m) / 30:2		
7. Increases FIO2 to 100%		
8. Checks IV-access		
9. Paused BLS < 15 seconds		
10. Correctly connects defibrillator < 2 min from circulatory arrest		
11. Identifies non-shockable rhythm		
12. Adrenaline (1 mg) is given IV in first block		
13. Rhythm check/pulsations every 2 minutes		
14. DD/ is named aloud using 4H/ 4T		
15. Clear division of roles, including team leader		

Total score	
Duration scenario	
Duration until maximum score	

Supplement S1 Scoring sheets of the critical management steps

Scenario VF without bundle

Session No.: **Investigator:**

Date: **Bundle:**

Act	Performed<br>0=no 1=yes<br>2=within time note	Remark
1. Controls pulsations within 1 minute		
2. Starts BLS within 1 min of detecting circulatory arrest		
3. Asks for help		
4. Starts timer		
5. Good quality BLS (5-6 cm deep, 100-120 x p/m) / 30:2		
6. Increases FIO2 to 100%		
7. Paused BLS < 15 seconds		
8. Correctly connects defibrillator < 2 min from circulatory arrest		
9. Identifies shockable rhythm		
10. Shock is delivered < 1 min after identifying shockable rhythm		
11. Three schock strategy used		
12. Amiodarone (300 mg) and Adrenaline (1 mg) is given IV on third shock		
13. Rhythm check/pulsations every 2 minutes		
14. DD/ is named aloud using 4H/ 4T		
15. Clear division of roles, including team leader		

Total score	
Duration scenario	
Duration until maximum score	

Supplement S1 Scoring sheets of the critical management steps

Scenario VF with bundle

Session No.:

Date:

Investigator:

Bundle:

Act	Performed<br>0=no 1=yes<br>2=within time note	Remark
1. Controls pulsations within 1 minute		
2. Starts BLS within 1 min of detecting circulatory arrest		
3. Uses bundle within 2 minutes		
4. Asks for help		
5. Starts timer		
6. Good quality BLS (5-6 cm deep, 100-120 x p/m) / 30:2		
7. Increases FIO2 to 100%		
8. Paused BLS < 15 seconds		
9. Correctly connects defibrillator < 2 min from circulatory arrest		
10. Identifies shockable rhythm		
11. Shock is delivered < 1 min after identifying shockable rhythm		
12. Amiodarone (300 mg) Adrenaline (1 mg) is given IV on third shock		
13. Rhythm check/pulsations every 2 minutes		
14. DD/ is called out loud using 4H/ 4T		
15. Clear division of roles, including team leader		

Total score	
Duration scenario	
Duration until maximum score	
